# Dynamic spatiotemporal patterns of brain connectivity reorganize across development

**DOI:** 10.1162/netn_a_00111

**Published:** 2020-02-01

**Authors:** Jakub Vohryzek, Alessandra Griffa, Emeline Mullier, Cecilia Friedrichs-Maeder, Corrado Sandini, Marie Schaer, Stephan Eliez, Patric Hagmann

**Affiliations:** Department of Radiology, University Hospital Centre and University of Lausanne, Lausanne, Switzerland; Department of Radiology, University Hospital Centre and University of Lausanne, Lausanne, Switzerland; Dutch Connectome Lab, Department of Complex Trait Genetics, Centre for Neuroscience and Cognitive Research, Amsterdam Neuroscience, VU University, Amsterdam, The Netherlands; Department of Radiology, University Hospital Centre and University of Lausanne, Lausanne, Switzerland; Department of Radiology, University Hospital Centre and University of Lausanne, Lausanne, Switzerland; Department of Neurology, Bern University Hospital, University of Bern, Switzerland; Department of Psychiatry, University of Geneva School of Medicine, Geneva, Switzerland; Department of Psychiatry, University of Geneva School of Medicine, Geneva, Switzerland; Department of Psychiatry, University of Geneva School of Medicine, Geneva, Switzerland; Department of Radiology, University Hospital Centre and University of Lausanne, Lausanne, Switzerland; Signal Processing Lab 5 (LTS5), École Polytechnique Fédérale de Lausanne, Lausanne, Switzerland

**Keywords:** Dynamic functional connectivity, Brain dynamics, Structural connectivity, Spatiotemporal connectome, Development, System diversity, Spatiotemporal diversity

## Abstract

Late human development is characterized by the maturation of high-level functional processes, which rely on reshaping of white matter connections, as well as synaptic density. However, the relationship between the whole-brain dynamics and the underlying white matter networks in neurodevelopment is largely unknown. In this study, we focused on how the structural connectome shapes the emerging dynamics of cerebral development between the ages of 6 and 33 years, using functional and diffusion magnetic resonance imaging combined into a spatiotemporal connectivity framework. We defined two new measures of brain dynamics, namely the system diversity and the spatiotemporal diversity, which quantify the level of integration/segregation between functional systems and the level of temporal self-similarity of the functional patterns of brain dynamics, respectively. We observed a global increase in system diversity and a global decrease and local refinement in spatiotemporal diversity values with age. In support of these findings, we further found an increase in the usage of long-range and inter-system white matter connectivity and a decrease in the usage of short-range connectivity with age. These findings suggest that dynamic functional patterns in the brain progressively become more integrative and temporally self-similar with age. These functional changes are supported by a greater involvement of long-range and inter-system axonal pathways.

## Author Summary

Maturation in human development is represented by changes in both functional dynamics and structural connectivity in the human brain. By constructing a spatiotemporal connectome for a cohort of 81 subjects ranging from 6 to 33 years of age, we demonstrate how these changes can be studied in a unified framework. We do so by defining two new measures of brain dynamics, namely the spatiotemporal diversity, mapping the level of temporal self-similarity of the functional patterns of brain dynamics, and system diversity, quantifying the level of integration/segregation between functional systems. These measures, we argue, represent a novel way of looking at brain dynamics constraints by structural connectivity. Using these measures, we show that dynamic functional patterns in the brain progressively become more integrative and temporally self-similar with age.

## INTRODUCTION

Human brain development from childhood to the early adult stage is marked by maturation of high-level brain processes, such as cognitive control, executive functions, and multimodal integration (Burr & Gori, 2012; Goswami, 2011). These processes arise through integration of information from local functional systems within complex neural networks, whose development is achieved through the optimization of white matter axonal bundles, as well as the selection of local connections at the neuronal level (i.e., synaptic pruning; Tau & Peterson, 2010). In order to characterize these neurodevelopmental processes, it is therefore of crucial interest to investigate the evolution of both structural white matter connections, as well as dynamic interactions between the different brain regions, as they give rise to mature and complex network organizations (Giedd & Rapoport, 2010; Hagmann, Grant, & Fair, 2012; Hwang, Hallquist, & Luna, 2013; Luna, Padmanabhan, & O’Hearn, 2010; Menon, 2013).

Network neuroscience serves as an ideal candidate to represent these relations in terms of brain static functional connectivity (FC), structural connectivity (SC), and, recently, dynamic functional connectivity (dFC). Both structural connectivity and functional connectivity have been widely studied (Collin & van den Heuvel, 2013). They depict various topological properties of large-scale brain architecture, such as small-worldness, hubness, and, in case of dynamic functional connectivity, network time-variance (Allen et al., 2014; Bullmore & Sporns, 2009; Grayson & Fair, 2017; Rubinov & Sporns, 2010). However, how dynamic functional connectivity patterns evolve with structural connections across neurodevelopment remains largely unknown.

The functional interactions among different brain regions in a resting state—an external stimulus-free condition—are often characterized as statistical dependencies between pairs of neural assemblies’ blood oxygen level–dependent (BOLD) signals. The modular structure of functional networks has been widely studied as they decompose into reproducible connectivity patterns, dubbed resting-state networks or functional systems (Damoiseaux et al., 2006; Fox et al., 2005; Yeo et al., 2011). Across human development, functional systems tend to be more and more integrated, thanks to the strengthening of long-range and inter-system functional interactions (DiMartino et al., 2014; Dosenbach et al., 2010; Fair et al., 2007; Marek, Hwang, Foran, Hallquist, & Luna, 2015; Menon, 2011; Vértes & Bullmore, 2015). Research on the dynamic aspects of these interactions has focused on exploring the temporal variability of functional connections’ strength during rest. Several studies have shown increased variability across time in functional connections, and particularly inter-system connections, with age (Hutchison & Morton, 2015; Marusak et al., 2017; Ryali et al., 2016). Yet, how dynamic interactions between structurally connected brain regions flexibly adapt and reorganize across time and ages is a topic of growing interest.

On the other hand, the structural brain network, mapping large axonal bundles, has been characterized by tracking water diffusion in brain white matter (Bullmore & Sporns, 2009; Hagmann et al., 2008). In development, small-world topology and all major white matter tracts are at place from an early age (Collin & van den Heuvel, 2013). Consequently, the developmental changes occurring across childhood and adolescence come from an adjustment of axonal diameters, myelination, and other factors (Hagmann et al., 2010). This leads to an overall maturation of white matter connectivity with profound effects on brain functions (Collin & van den Heuvel, 2013; DiMartino et al., 2014; Dumontheil, 2016; Hagmann et al., 2012; Vértes & Bullmore, 2015). Indeed, the correlation between structural and functional connectivity values in the brain tends to increase from toddler age to late adolescence, suggesting a consolidation of functional patterns coherent with the maturation processes of the underlying white matter substrate (Hagmann et al., 2010). With growing evidence of functional connectivity patterns being shaped by the structural connectivity architecture, an interesting question arises as to how the underlying white matter structure affects the dynamics of functional connections with age.

The aim of this work is to investigate brain changes of dynamic functional connectivity interactions, and their relationship with the underlying white matter maturation processes from early childhood to late adolescence and young adulthood. To this end, we represent the structural and functional connectivity information of individual healthy subjects, from 6 to 33 years of age, using a previously proposed multilayer graph framework, named spatiotemporal connectome (Griffa et al., 2017). This network representation allows decomposition of the fMRI data into time-varying functional coactivation patterns between anatomically connected brain regions. This brings the functional and structural network-based investigation of brain development under one unified framework (Griffa et al., 2017). By doing so we were able to characterize, in a combined fashion, the maturation of functional and structural connections in terms of (a) the integration and segregation dynamics of the different functional systems, (b) the spatiotemporal heterogeneity of functional coactivation patterns, and (c) the functional role of long-range inter-system versus short-range structural connections. In such a way, we tested the hypothesis that dynamic functional activity becomes functionally more integrative across development and at the same time results in consolidated activity patterns with higher spatiotemporal stability.

**Figure 1.**
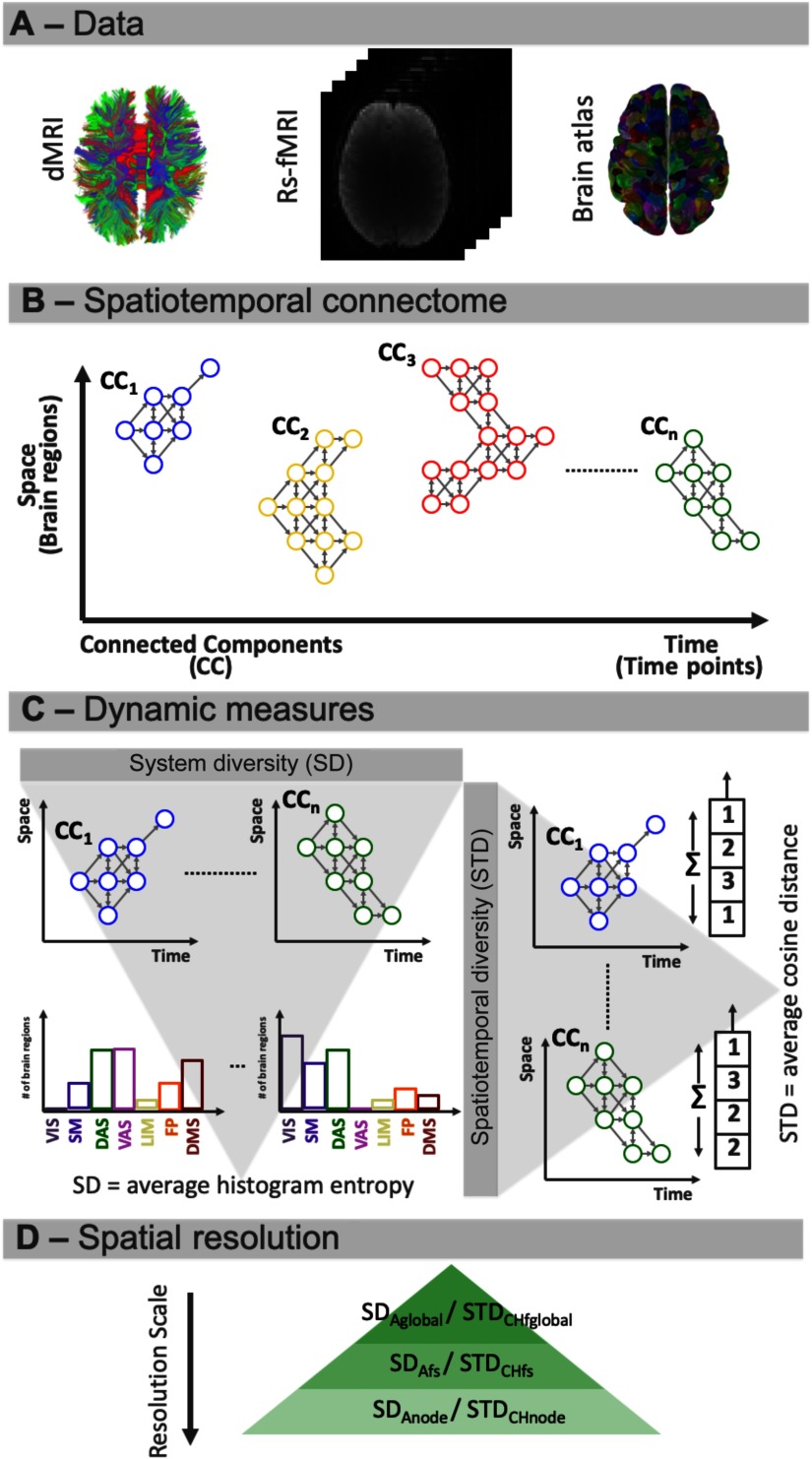
Computation of system diversity and spatiotemporal diversity. (A) Data used. (B) From the BOLD signal of 448 brain regions and template structural connectome, we constructed a sparse multilayer graph represented in the form of connected components. (C) We concatenated all subjects within a given age group and calculated the SD and STD measures. SD is computed as functional system probability distribution entropy and is representative of the functional systems’ distribution for each connected component. Functional systems were defined as visual (VIS), somato-motor (SM), dorsal attention (DA), ventral attention (VA), limbic (LIM), frontoparietal (FP), and default mode (DM) systems (Yeo et al., 2011). STD is measured as an average cosine similarity between vector-embedded connected components and tracks the dynamic spatiotemporal versatility of the individual brain regions. (D) Both SD and STD between groups were calculated (a) globally, (b) for CCs of specific functional systems, and (c) for CCs involved in individual brain regions.

To be able to test this hypothesis, we developed two new measures to characterize the functional role of brain regions in a spatiotemporal connectome (Figure 1). First, we constructed spatiotemporal connectomes of individual subjects, which allowed us to map the dynamic patterns of functional coactivation constrained by structural connectivity (Figures 1A, B). Formally, these dynamic functional patterns correspond to the connected components of the multilayer network. Second, to reflect the spatiotemporal heterogeneity of the dynamic patterns, we defined a spatiotemporal diversity (STD) measure quantifying the cosine-similarity between pairs of vector-embedded connected components (Figure 1C). Third, to address dynamic integrative properties between brain functional systems, we introduced a system diversity (SD) measure based on the entropy of the histogram of functional systems’ distribution for each connected component (Figure 1C). Using these methodological instruments, we investigated, across multiple scales of analysis, how brain spatiotemporal dynamics mature with age, in terms of inter-system integrative capacity, dynamic patterns’ heterogeneity, and long/short range structural connections’ usage (Figure 1D).

## RESULTS

From resting-state functional MRI (fMRI) data and a cohort-template structural connectivity matrix, we constructed the spatiotemporal connectomes (Griffa et al., 2017) for 69 subjects from 6 to 33 years of age (see the Supporting Information). We used a cortical parcellation of 448 brain regions from the Lausanne 2008 atlas (Cammoun et al., 2012). We extracted the multilayer graph representation of functional coactivation patterns between anatomically connected brain regions, as series of weakly connected components (CCs; Griffa et al., 2017). See Figures 1A and 1B. Moreover, we associated with every brain region a functional system (FS) as defined by Yeo et al. (2011): visual (VIS), somato-motor (SM), dorsal attention (DA), ventral attention (VA), limbic (LIM), frontoparietal (FP), and default mode (DM) systems. Furthermore, we defined two novel measures—namely, the spatiotemporal diversity (STD) and the system diversity (SD)—to characterize the functional system dynamic recruitment and temporal self-similarity of the different CCs (Figure 1C). Both measures were compared between two age groups—adults (median [interquartile range] = 24.3 [6.7]) and children (median [interquartile range] = 10.7 [2.8])—across three different spatial scales of investigation: global, functional system level, and nodal level (see Figure 1D and the Methods section). Last, we examined the connected components’ properties on the edge level, in relation to the white matter fiber length and inter-system connectivity. For details about the number, spatial, and temporal size of the CCs in relation to age, see the Supporting Information.

### Spatiotemporal Connectome Measures

For the developmental analysis, two new measures of the CCs were defined, namely the system diversity (SD) and the spatiotemporal diversity (STD). From an intuitive point of view, every brain region will be recruited in several CCs across an fMRI recording. These CCs can vary in their association with the different functional systems (Yeo et al., 2011), and in their spatiotemporal similarity. These two features are described by the SD and STD measures, respectively. The SD quantifies the heterogeneity of functional systems’ allegiance within an individual CC and is computed as the entropy of the functional system probability distribution across all unique regions recruited within the CC (Figure 1C). SD represents the integrative properties of each individual CC. STD, on the other hand, quantifies the spatiotemporal diversity of CCs, and it is therefore defined for pairs of CCs. The STD is measured as the average cosine similarity between vector-embedded CC pairs (Figure 1C). The STD tracks the spatiotemporal versatility of the dynamic functional patterns represented by the CCs. We calculated these two measures for the adult and children groups, for three distinct resolution scales of investigation. The investigated scale defined what subset of CCs was considered for the group comparison: global scale—all CCs for a particular age group; functional system scale—all CCs with at least 20*%*of brain regions part of a functional system, for a particular age group; and node scale—all CCs involving a specific brain region, for a particular age group (see Figure 1 and the Methods section).

### Global Reorganization of Spatiotemporal Patterns With Age

**Figure 2.**
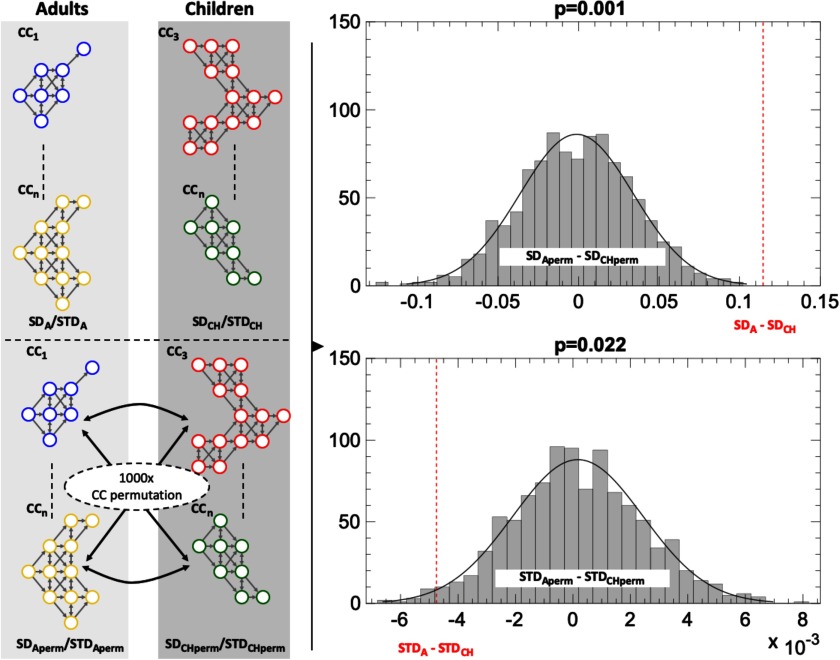
SD and STD at the global level. Comparison of SD and STD between the two age groups of adults (A) and children (CH). The left diagram represents the real and permuted values of both measures. The right diagram shows the null distributions as a difference between the two groups in relation to the real difference between the two groups (SD: *p* value = 0.001 and STD: *p* value = 0.022, two-sided permutation test).

First, we tested the hypothesis that from childhood to adulthood, the brain develops functionally integrated dynamic patterns, while at the same time becomes more spatiotemporally conservative in their recruitment. To this end, we quantified the difference in STD and SD values between the children and adult groups, on the global level of investigation. As the measures are defined on a group level, the difference between the average STD and SD values over all the CCs in each age group was statistically evaluated in relation to a null distribution computed from 1,000 permutations of CC reassignment between the two age groups. For a group comparison between children (median [interquartile range] = 10.7 [2.8]) and adults (median [interquartile range] = 24.3 [6.7]), the global SD was found to be larger in adults than in children (*p* = 0.001, two-tailed permutation test, Figure 2), suggesting more integrative spatiotemporal patterns in adults. On the other hand, STD was decreased in adults compared with children (*p* = 0.022, two-tailed permutation test, Figure 2), suggesting a decrease in the repertoire of spatiotemporal patterns with age.

### Function-Specific Reorganization of Spatiotemporal Patterns With Age

**Figure 3.**
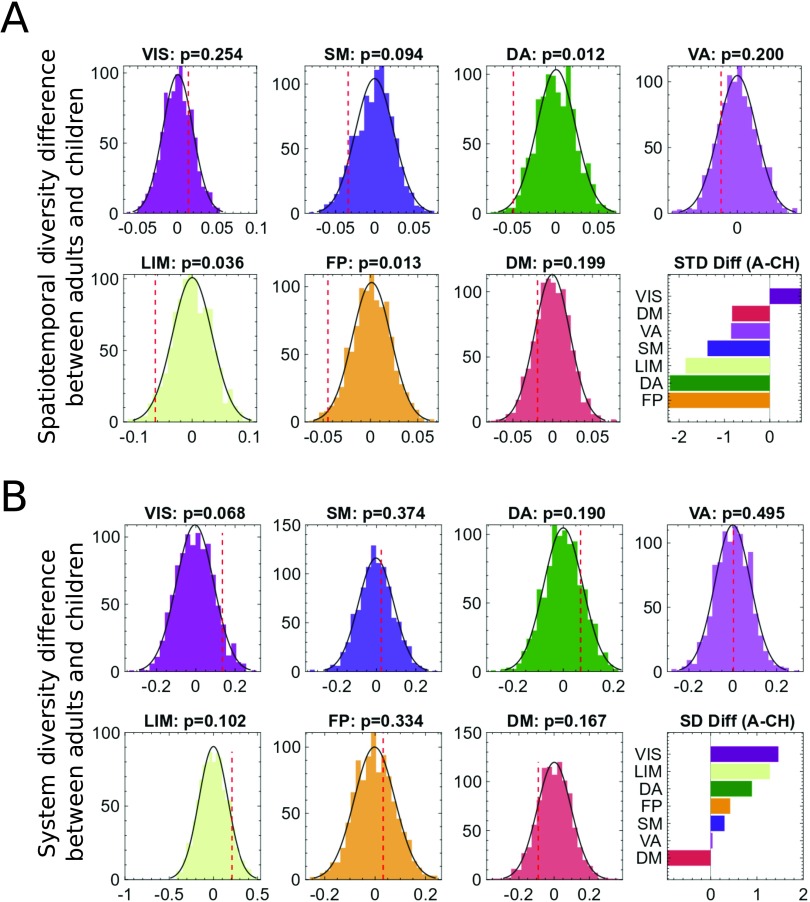
Spatiotemporal diversity (STD) and system diversity (SD) at functional system level. Comparison of SD and STD between the two age groups of adults (A) and children (CH) for individual functional systems between the real value (dashed red line) and its null distribution. (A) STD for individual functional systems between the groups (STD, frontoparietal (FP): *p* value = 0.021, dorsal attention (DA): *p* value = 0.024, limbic (LIM): *p* value = 0.031, one-tailed permutation test FDR corrected). (B) SD for individual functional systems between the groups.

To further characterize the maturation of functional dynamics with age, we extended the described global analyses to the functional system level. For each functional system *i*, we carried out a permutation test considering only the CCs related to system *i*. We defined a CC to be related to the functional system *i* if at least 20% of the cortical regions active in that CC belong to system *i*, according to the functional parcellation defined in Yeo et al. (2011) See Figure 3. We observed significant STD differences between children and adults for the frontoparietal, dorsal attention, and limbic systems (FP: *p* = 0.021, DA: *p* = 0.024, LIM:*p* = 0.031, one-tailed permutation test FDR corrected, Figure 3, top). The different functional systems can be related to higher order (FP, LIM, VA, and DA) and lower order (VIS and SM) systems, and default mode systems (Margulies et al., 2016). Along this line of reasoning, our results suggest that the consolidation of functional activation patterns and related structural communication pathways from childhood to adulthood mainly happens at the level of the higher order cognitive systems, with the exception of the VA system. When investigating possible age-related changes of SD values at the level of the single functional systems, we did not observe any statistically significant difference between the children and adult groups, for any of the functional systems. Considering the fact that SD values were higher in adults than in children at the global level, this result indicates a global, but not system-specific, tendency of increased functional integration with age (Figure 3, bottom).

### Nodal Dynamic Cortical Signature of Spatiotemporal Patterns in Different Age Groups

**Figure 4.**
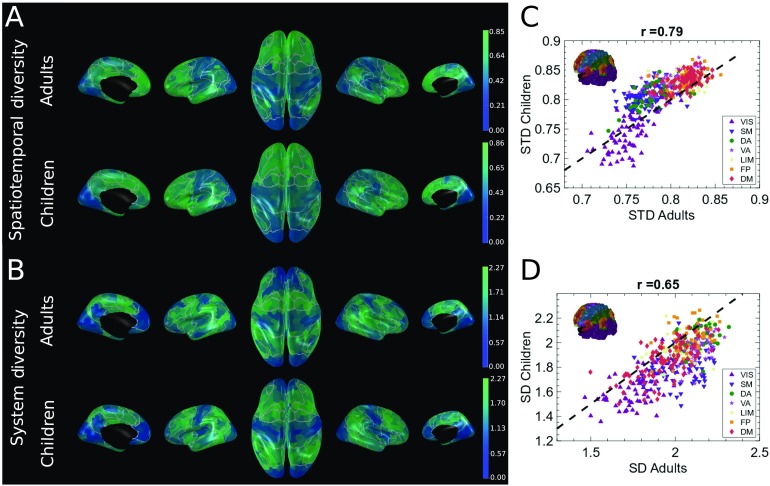
Cortical patterns of spatiotemporal diversity (STD) and system diversity (SD) values in children and adults. Every brain map represents values of STD and SD for the 448 cortical nodes. (A) Cortical maps for STD of the adults and children groups. (B) Cortical maps for SD of the adults and children groups. Correlation plots represent the relationship between the STD and SD values of the two groups. Dashed black line stands for the identity line, with different colors representing different functional systems: visual (VIS; purple), somato-motor (SM; blue), dorsal attention (DA; green), ventral attention (VA; pink), limbic (LIM; yellow), FP (orange), default mode (DM; red). White demarcation lines represent the separation of the seven functional systems. (C) Correlation between STD adults and STD children (*r* = 0.79). (D) Correlation between SD adults and SD children (*r* = 0.65). The inserted brain in the correlation graphs represents the Yeo functional systems (Yeo et al., 2011).

Next, we examined both the STD and the SD measures at the nodal level to characterize the dynamic properties of the different brain regions, and we qualitatively compared the derived cortical maps between children and adult groups. For the STD in the adult group (Figure 4, top left), we observed a clear separation between regions belonging to primary lower order functional systems (VIS and SM) and higher order systems (FP, LIM, and VAS), with the exception of the DA system having similar values as the lower order systems. Higher order areas depict more heterogeneous spatiotemporal functional patterns (higher STD) compared with primary areas, possibly reflecting their functional versatility and lower degree of specialization. Similar demarcation between higher order cognitive systems and lower order areas was also consistent in the children group (Figure 2, top left). However, children had globally more heterogenous spatiotemporal patterns compared with the adult group, as indicated by the distribution of the nodal STD values above the 45-degree equality line in the adult-children nodal-STD scatterplot (Figure 4, top right). On the other hand, children demonstrated globally lower SD values compared with adults (see adult-children nodal-STD scatterplot, Figure 4, bottom right), indicating lower levels of integration between the different functional systems. The cortical patterns of SD values were similar between the two groups (Figure 4, bottom left). For both children and adults, regions belonging to the default mode system demonstrated unique spatiotemporal features with high STD but low SD, suggesting that default mode spatiotemporal activation patterns are highly heterogeneous in time, with few interactions with the other functional systems.

### Brain Integrative Tendencies in Development

To test the hypothesis that the brain shifts from more localized brain activity patterns to global representations across development, we investigated the relationship between the subjects’ age, and the functional recruitment of structural connections with different physical lengths. We note that, in our framework, each edge of the multilayer graph represents a functional coactivation of two brain regions structurally connected through a white matter bundle of a certain spatial length. We associated with each edge of the spatiotemporal connectome a physical length, as derived from a 68-subject structural connectivity template (see the Supporting Information). We chose two length-thresholds to classify the network edges as short-range or long-range, namely 20 mm and 42 mm, consistent with the literature (Baker et al., 2014; Behrman-Lay et al., 2015). See Figure 2.

**Figure 5.**
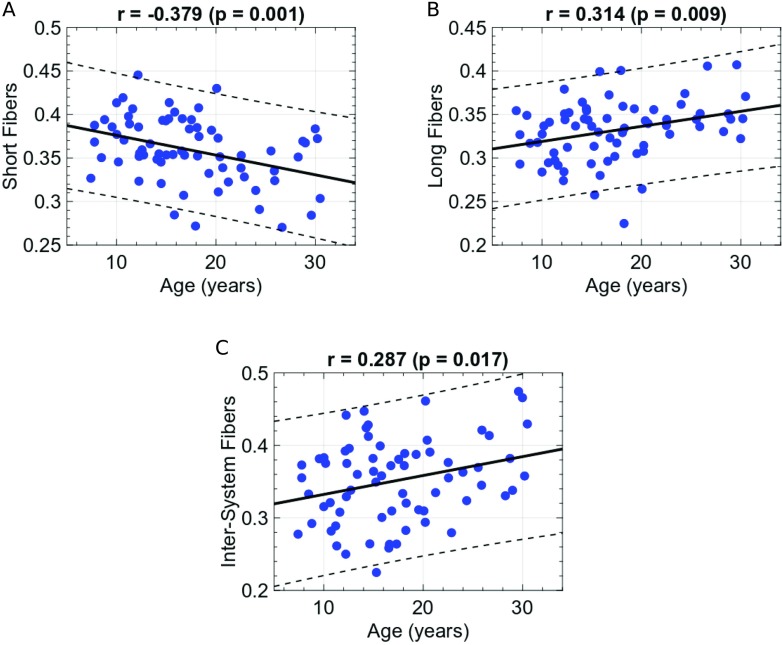
Development of brain integration. (A) Changes in relative use of short edges (to the subject’s maximum number of edges used) as a function of age. (B) Changes in relative use of long edges (to the subject’s maximum number of edges used) as a function of age. (C) Changes in relative use of intra-system edges (to the subject’s maximum number of edges used) as a function of age.

As each subject will have a unique multilayer network with a varying number of connected components and edges by construction, we normalized the number of short/long edges of each subject’s spatiotemporal connectome by the total number of edges for each subject and reported the relative number of short/long edges per subject. This approach allowed us to quantify the relative (and not the absolute) functional usage of structural connections with age. From childhood to early adulthood, we found a significant positive association between age and the probability of long-range neural pathways functional usage (> 42 mm, Pearson’s*r* = 0.31, *p* = 0.009, significant for 42 ± 5-mm-length threshold, Figure 5B, and a negative association between age and short connections functional usage (< 20 mm, Pearson’s *r* = −0.38, *p* = 0.001, significant for 20 ± 5-mm-length threshold, Figure 5A). These correlations remained significant after including the average frame-wise displacement as a nuisance regressor (long edges: Pearson’s *r* = 0.25, *p* = 0.038; and short edges: Pearson’s *r* = −0.33, *p* = 0.005; Power, Barnes, Snyder, Schlaggar, & Petersen, 2012).

Next, we focused on how the integrative tendencies play out in the redistribution of edges between different functional systems. To this end, we assigned to each edge of the multilayer graph a label indicating whether that edge represents an inter- or intra-system connection. The functional systems’ labels were taken from the FSs described in Yeo et al. (2011). We normalized each subject’s edge distribution by the total number of edges present in the subject’s spatiotemporal connectome. Thus, we looked at the relative (and not absolute) use of the inter-system and intra-system edges. We showed that the probability of recruiting inter-system edges increases with age (Pearson’s *r* = 0.29, *p* = 0.017, Figure 5C). Also, the results remained significant after regressing out possible motion artefacts, quantified by the average frame-wise displacement (Pearson’s = 0.015; Power et al., 2012). These results indicate that the functional usage of long-range, inter-system axonal pathways increases from childhood to early adulthood and is in line with the consolidation of more integrative functional dynamics shown by the above-described SD and STD analyses. During development, the human brain reconfigures toward more integrative, large-scale, and inter-system functional interactions.

## DISCUSSION

In this work, we investigated the developmental changes of functional coactivation patterns constrained by the underlying structural connections, in a cohort of 69 subjects aged between 6 and 33 years. Our results demonstrate that, from childhood to adulthood, the brain spatiotemporal dynamics undergo a functional reorganization, where brain regions are recruited in more functionally heterogeneous patterns of activity with age. Moreover, these patterns of activity grow to be more spatially and temporally consolidated with age. These results are supported by further analyses on the increasing usage of long-range as well as inter-system white matter connections. Our findings corroborate previous developmental studies that focused on functional and structural connectivity maturation independently. These studies indicate that structural networks mature from spatially proximal to a more integrative topology, supporting functional reorganization of increasingly complex higher order cognitive interactions between various resting-state networks (Collin & van den Heuvel, 2013; Grayson & Fair, 2017; Hagmann et al., 2012; Vértes & Bullmore, 2015).

### Dynamic Reorganization of Brain Activity Patterns

Our analysis on spatiotemporal patterns shows that global SD increases with age, whereas global STD decreases with age. This suggests that brain activation becomes increasingly functionally distributed, while becoming more consolidated in its spatiotemporal configurations. Indeed, the result on SD supports an image of functionally collaborative brain regions, engaging in complex cognitive functions with age (Marusak et al., 2017; Vértes & Bullmore, 2015). On the other hand, the decrease of STD with age seems to corroborate previous literature on static FC when looking at the dynamic aspects of FC. Functional networks have been shown to have rudimentary topological architecture, such as functional hubs and well-defined resting-state networks, at place early on in development, with major changes coming from strengthening of cross-domain and hub-spoke region connections (Hwang et al., 2013; Marek et al., 2015). From a dynamic perspective, this is in congruence with the functional usage of increasingly more mature and conservative channels of communication, potentially leading to effective information processing necessary for mature cognitive functioning.

The STD changes (decrease) with age were mainly driven by higher order areas, including the frontoparietal and dorsal attention systems, suggesting a prominent consolidation of these functional systems across the age range investigated in this study (Fair et al., 2007; Supekar, Musen, & Menon, 2009). When considering the cortical maps of the STD and SD values, a particular position is occupied by the regions belonging to the default mode system—a task-negative system associated with inner thinking, representation of self, and internal modes of cognition (Raichle et al., 2001). DM regions including the precunei, medial prefrontal cortices, and angular gyri simultaneously demonstrate low SD values consistent with the notion of task-negative network (Karahanoğlu & Van De Ville, 2015; Raichle, 2015) and high STD, in line with recent literature suggesting the existence of diverse spatiotemporal DM patterns in dynamic functional connectivity analyses (Griffa et al., 2017; Karahanoğlu & Van De Ville, 2015). It is worth noting that in our analyses the default mode system, as opposite to the other functional systems, tended to have lower SD values in adults compared with children (although this difference was not statistically significant; Figure 3), possibly suggesting a consolidation of the task-negative role of this network across neurodevelopment.

### Integrative Age-Related Changes in Edges of the Spatiotemporal Connectome

Our results showed distance and inter-system age-related changes of structural connections usage. The probability of functional recruitment of long-range white matter connections increased with age, whereas the probability of recruiting short-range connections tended to decrease with age. These findings are in line with previous results on static functional connectivity strength, which demonstrate an increase for distant and a decrease for proximal region pairs (Fair et al., 2007; Supekar et al., 2009; Vértes & Bullmore, 2015). However, these findings were recently put into question because of concerns about possible effects of motion artefacts, which might lead to an overestimation of distance-dependent functional effects (Grayson & Fair, 2017; Marek et al., 2015). It is also important to point out that previous studies used Euclidean distances to characterize the maturation of functional connectivity strength in relation to brain regions’ spatial relationships. In contrast, our method takes into account only functional relationships between region pairs that are structurally connected by white matter axonal pathways, thus adding to the refinement and robustness of our findings.

The probability of inter-system connection functional usage increased with age. These findings support a picture of brain functional systems collaboratively engaging in tackling higher order cognitive functions with age (Marek et al., 2015; Vértes et al., 2012). Previous studies, which focused on the modular evolution as a defining feature of brain functional integration, are however at odds with these results, as they observe a global decrease in inter-system and a global increase in intra-system functional connectivity, suggestive of formation of functional modules rather than their refinement and collaborative tendencies between them (Gu et al., 2015; Stevens, Pearlson, & Calhoun, 2009). This discrepancy might very well result from the approach taken to describe the brain communities, as all the mentioned studies concentrate on the static FC description of brain activity. However, when looked at from the dynamic perspective, in the sense of communities being defined along not only spatial dimension but also temporal, it is possible to gain more realistic insight into the brain communities’ organization in development. Thus when drawing from our results on ST and STD, (i.e., system diversity increases with age whereas the diversity of spatiotemporal patterns decreases with age, mainly driven by FP, DA, and limbic systems), it is possible to observe the brain network development as a tendency toward increasing interactive system with increasing specialization of their functioning.

### Conceptual Differences Among dFC Methods

In general, there is still no consensus about the conceptualization of brain dynamics. This is also the case with respect to resting-state fMRI time-dependent analyses, where currently proposed methods are still in their early days. Originally, brain dynamics were described by static functional connectivity (Bullmore & Sporns, 2009). Recent methods have focused on representing brain dynamics as sequences of discrete states of brain activity changing over time (Allen et al., 2014; Baker et al., 2014). These methods deliver dFC measures such as fractional occupancy (the fraction of time a state remains active over a recording period), lifetime of states (how long on average a state lasts in a sequence), or switching probability (how frequently individual states switch between them). These approaches have also been implemented in development studies in case of static FC (Betzel et al., 2014; Fair et al., 2007) and state-based dFC (DiMartino et al., 2014; Hutchison & Morton, 2016; Ryali et al., 2016). However, how individual nodes engage in temporally diverse (STD) and rich (SD) transient patterns of activity is another way to conceptually regard the brain dynamic interactions (Griffa et al., 2017). In this new perspective, we are not looking at a static functional connectivity or state-based dFC, but rather at nodal dynamics constrained by structure. As such, STD can be regarded as quantifying the versatility of nodal broadcasting, and SD as temporal diversity of nodal broadcasting with respect to the different functional systems. From the visual inspection of the adults’ brain maps (Figure 4), we can observe that the frontoparietal system, responsible for coordinating behavior in a rapid, accurate, and flexible way, has high STD values, suggesting a high versatility of nodal broadcasting in these areas. Future work should therefore explore how the values of STD relate to behavioral measures of cognitive flexibility and/or creativity. In the same line of thinking, adults’ brain maps (Figure 4) present high SD values in associative areas, indicating that regions responsible for sensory integration and information broadcasting toward higher order systems tend to coactivate with multiple functional systems in time. Future work should relate the values of SD to the multisensory integration tasks.

### Spatiotemporal Connectome and Dynamic Measures in the Brain

Describing brain dynamics has been of growing interest in the neuroscience community, with several studies focusing on dynamic FC features in neurodevelopment. The SD measure describes the functional heterogeneity of connected components in terms of functional system cooperation, and can be related to studies investigating the temporal variability of functional connectivity strength (Chai et al., 2017; T. Chen, Cai, Ryali, Supekar, & Menon, 2016; Hutchison & Morton, 2015; Marusak et al., 2017). On the other hand, the connected components of the spatiotemporal connectome map the functionally and structurally relevant connections in the brain over time. The level of spatiotemporal diversity of the connected components is quantified by the STD measure. In this sense, STD relates to previously introduced “flexibility” measures quantifying the temporal variability of community structure in multilayer graphs with a temporal dimension (Bassett et al., 2013). However, none of the aforementioned studies consider the structural connectivity constraints of brain dynamics as included in the spatiotemporal connectome.

### Methodological Considerations

We chose the spatiotemporal connectome as an investigation framework, since it provides an original perspective on different research questions by unifying structural and functional information under one umbrella. It is to be noted that the framework suffers from several methodological limitations. The diffusion MRI data reconstruction and tractography ultimately introduce false positive and negative structural connections, requiring the use of a group structural template (de Reus & van den Heuvel, 2013). This however made the subject-wise analysis of the structural connectomes difficult to implement in the framework. Furthermore, the functional coactivation patterns were derived from a point-process analysis of the fMRI signals (Tagliazucchi, Balenzuela, Fraiman, & Chialvo, 2012), which is a simple assumption stating that a neuronal event happens at the significant peak of BOLD activity. This choice makes the final spatiotemporal connectome sensitive to the choice of the point-process threshold. However, it has been shown that this very sparse signal representation is able to preserve the salient statistical aspects of the fMRI signal (Tagliazucchi, Siniatchkin, Laufs, & Chialvo, 2016).

The construction of the spatiotemporal connectome constrains the time-resolved coactivations only to the anatomically connected brain regions. While it has been shown that this implicit hypothesis is related to temporal stability of functional dynamics as well as to the most reproducible functional interactions (Griffa et al., 2017; Shen, Hutchison, Bezgin, Everling, & McIntosh, 2015), it doesn’t take into consideration cases where time-resolved coactivations may arise between disconnected regions. This information is of course relevant in describing the full picture of the time-resolved coactivations, but the occurrence of those events is significantly less prevalent than in the connected case—it has been shown to be on average 12 times less likely to obtain coactivation between anatomically disconnected brain regions to the anatomically connected brain regions (Griffa et al., 2017). However, extra caution should be exercised when trying to interpret SD/STD results in specific brain regions, which tend to possess relatively high coactivations in the absence of anatomical connections, as shown previously for bilateral auditory cortices or the lateral postcentral gyri, for example. On one hand, these temporal coactivations might result from synchronization of external stimuli and thus require different interpretations for their occurrence. On the other hand, this could simply be due to the inability to fully map difficult fiber tracks such as the commissural fibers. Further studies could look into these specific areas (Griffa et al., 2017; Thomas et al., 2014). This in fact is one of the methodological shortcomings, as dMRI tractography is known to suffer from both image acquisition and algorithmic problems (Jones, Knösche, & Turner, 2013), and by default the yielded structural matrices obtain a proportion of false positive and false negative connections (de Reus & van den Heuvel, 2013). In future work, the spatiotemporal framework could be improved by adding probability of existence to every structural connection (Daducci, Dal Palú, Descoteaux, & Thiran, 2016; Hinne, Heskes, Beckmann, & van Gerven, 2013).

Recently, the problem of motion artefacts has been debated in relation to the decreased statistical significance of some of the neurodevelopmental findings reported in previous literature (Grayson & Fair, 2017). According to these reports, head motion tends to increase local functional coupling and decrease long-range functional connectivity, which directly relates to some of the findings in neurodevelopment (Grayson & Fair, 2017; Power et al., 2012; Satterthwaite et al., 2012). There is an ongoing debate on the influence of the motion artefacts in fMRI studies (Power, Schlaggar, & Petersen, 2015). Various approaches have been proposed to attenuate motion artefacts, involving the usage of motion nuisance regressors, global signal regression, wavelet despiking, or motion censoring (Patel & Bullmore, 2016; Power et al., 2015; Siegel et al., 2014). In light of these concerns, and in addition to the standard motion correction procedures (see Methods and the Supporting Information), we applied a motion-correction strategy tailored to fMRI point-process analysis: that is, the censoring (or scrubbing) of time points possibly associated with motion (Carhart-Harris et al., 2014; Siegel et al., 2014; Tagliazucchi et al., 2016). Moreover, we applied a stringent scrubbing correction by eliminating all the connected components contained in at least one time point associated with motion (as opposed to simple individual time point scrubbing as done in standard point-process analysis; see the Supporting Information). Finally, motion-derived signals were taken into account in regression analyses.

## Conclusion

In summary, we developed two novel measures to characterize how brain functional dynamics evolve across neurodevelopment, from childhood to young adulthood. Our study suggests that the brain in the adult population becomes more functionally integrative, as described by the increased global SD values with age, while reducing its repertoire of brain dynamics, as shown by the decreased global STD values with age. Although the aforementioned measures are only two ways of describing the complex dynamics of brain development, they provide an original conceptual approach to investigating the dynamic diversity of functional patterns, in relation to well-known functional systems, as well as in relation to the temporal heterogeneity and consolidation of the brain spatiotemporal organization. Understanding the dynamical aspects of brain functional reorganization, taking place from childhood to adulthood, can not only help elucidate fundamental mechanisms of brain development, but also improve our understanding and early diagnosis of neurodevelopmental disorders. It is the authors’ hope that this study could be a step in this direction.

## Methods

### Participants

A total of 81 participants (101 data points) were considered for this developmental study. The cohort’s age ranged from 6 to 33 years of age (median [inter-quartile range] = 16.2 [9.0]; see the Supporting Information). Each subject underwent an MRI session on the 3 Tesla Siemens Trio scanner at the Centre d’Imagerie Biomédicale (CIBM) in Geneva, Switzerland. The MRI session consisted of three acquisition protocols: (a) a structural MRI acquired with T1-weighted contrast (0.9 × 0.9 × 1.1 mm voxel resolution, TR = 2,500 ms, TE = 3 ms, TI = 1,100 ms, acquisition matrix = 256 × 256, 192 slices, flip angle = 8°); (b) a diffusion MRI (dMRI) acquired with diffusion tensor imaging (DTI) sequence (2 × 2 × 2 mm voxel resolution, TR = 8,800 ms, TE = 84 ms, flip angle = 40°, 30 directions and maximum b = 1,000s/mm^2^), acquisition matrix = 128 × 128,64 axial slices and slice thickness = 2 mm; and (c) a resting-state functional MRI (fMRI) acquired for 8 min (2.4 × 1.8 × 3.2 mm voxel size, TR = 2,400 ms, TE = 30 ms, 38 slices, flip angle 85°). During the fMRI acquisition, subjects were asked to not fall asleep and let their mind wander while fixating their vision to the cross on the screen. All participants provided a written consent, and the study was approved by the Institutional Review Board of the Geneva University School of Medicine.

### Structural Connectivity Template

In the spatiotemporal connectome, we used a template structural connectivity matrix derived from a cohort of 68 subjects with diffusion spectrum imaging (DSI) acquisition. This structural template has already been used in previous works (Griffa et al., 2017). In brief, the subjects’ structural MPRAGE volumes were delineated into white matter, gray matter, and cerebrospinal fluid using FreeSurfer software, v.5.0.0. Then, the DSI dataset was reconstructed and the orientation distribution function (ODF) was estimated in each voxel (Wedeen, Hagmann, Tseng, Reese, & Weisskoff, 2005). With the upper limit of three, the main fiber orientation was considered as the largest maxima of the ODF in each voxel. Furthermore, deterministic streamline tractography (Jones, 2008), with 32 streamline seeds per white matter voxel and per fiber orientation, was performed. A boundary-based cost function (FreeSurfer) was used to register the subject diffusion space (b0) to the MPRAGE and brain parcellation (Greve & Fischl, 2009). Morphological brain parcellations and the diffusion data were taken together to obtain subject-wise structural connectivity matrices. Thanks to the inclusion of high b-value diffusion encodings, DSI data provide a more superior angular resolution of local diffusion profiles than low b-value DTI data (Wedeen et al., 2008). DSI-based tractography can therefore resolve complex fiber configurations and deliver a richer whole-brain tractogram and structural connectivity information. For this reason, we chose this external structural connectivity template to build the spatiotemporal connectomes of the children and young adults included in this study. The structural template was constructed as a binary network where connection was considered if at least 50% of the subjects possessed it (see the Supporting Information).

### MRI Processing

The T1-weighted image segmentation delineated the white matter, gray matter, and cerebrospinal fluid. Subsequent parcellation of the cortical mantel into 448 brain regions of interest was done according to Cammoun et al. (2012), with subcortical structures excluded from the analysis. The fMRI dataset was slice-time corrected and motion corrected using rigid-body coregistration (Jenkinson, Bannister, Brady, & Smith, 2002). The scrubbing parameters, frame-wise displacement (FD), and DVARS were computed prior to further preprocessing steps (Power et al., 2012, 2014). Special care was taken of the motion-induced artefacts, with an additional 14 scans excluded because of a number of motion-corrupted time frames exceeding 10% of the total scanning time, for thresholds of FD = 0.4 mm and DVARS = 25. Additionally, a novel scrubbing technique was performed in the multilayer graph space, where we discarded all connected components implicated in motion-corrupted time frames, thus excluding any potential functional activations that might have arisen several time points before or after the corrupted time frame. (See the Supporting Information for further details on motion correction.) Next, in order to allow signal stabilization, the first four time frames were excluded. Voxel-wise signals were detrended and motion corrected, and physiological confounds were minimized by regressing the average white matter and cerebrospinal fluid (CSF) signals, and the six motion signals (three translational and three rotational). The Hamming windowed-sinc FIR filter was used to bandpass filter the preprocessed fMRI series (0.01–0.1 Hz). For each subject, the T1-weighted volume and the derived brain cortical parcellation were linearly registered to the mean fMRI volume (FSL software; Jenkinson, Beckmann, Behrens, Woolrich, & Smith, 2012), and an average fMRI signal was computed for each one of the 448 cortical regions. The MRI preprocessing was performed in the subject space with the Connectome Mapper (Daducci et al., 2012) and Matlab and Python scripts.

### Spatiotemporal Connectome Construction

The preprocessed fMRI signal and the SC template were combined into a unified framework previously named spatiotemporal connectome (Griffa et al., 2017). In detail, the fMRI time series were z-scored and thresholded at 2 standard deviations in order to obtain a binary point-process for each brain region (Tagliazucchi et al., 2012). A region-wise point-process is a binary sequence indicating the active (z-scored fMRI signal above the threshold) and quiescent (z-scored fMRI signal below the threshold) time points for that region. For a choice of the threshold, please refer to Griffa et al. (2017). Connections of the multilayer graph, $\tilde{G}$, were created if two brain regions were both functionally coactive and structurally wired (according to the above-described structural connectivity template). This has been performed for connections taking place at the same time and across two successive temporal frames. By doing so, the multilayer graph encoded weakly connected components (CCs; Kivelä et al., 2014) representing the functional spreading of coactivation patterns on the structural brain scaffold.

### Spatiotemporal Connectome Measures

Every CC of the multilayer graph $\tilde{G}$ can be thought of as encoding functional activation of particular brain regions represented in time and space. Here, a particular CC is described as a spatial activation map by defining a vector *x* = [*x*_1_,*x*_2_,…,*x*_*N*_], where *N* is the number of brain regions and *x*_*i*_ represents the number of time points within a CC a brain region *i* is active for. The spatial activation map is normalized by the vector l2-norm as $\overset{-}{x}=\sqrt{\sum_{i\text{=1}}^{N}{\left|x_{i}\right|}^{\text{2}}}$. By characterizing the CCs using their spatial activation vectors, we can define two new measures of brain dynamics, namely SD and STD.

We defined the system diversity as a functional system histogram-based entropy measure. First, we associated with each entry of the vector $\overset{-}{x}$ a functional system label *f* (according to the classification of Yeo et al., 2011). Second, we built for each *x* a probability vector $\overset{-}{p}=[p_{1},p_{2}\text{,…,}p_{FS}]$, where *FS* is the number of functional systems and *p*_*f*_ represents the number of regions within a CC labeled as belonging to a specific functional system *f*. Then, we computed the functional system histogram-based entropy as $\text{SD}=\sum_{f\text{=1}}^{FS}-P(p_{f})\text{log}P(p_{f})$, where *P*(*p*_*f*_) is the probability mass function.

We defined the spatiotemporal diversity as the average cosine similarity between the spatial activation maps of CC pairs as ${STD}_{scale}=\frac{\text{2*}\sum_{n\text{=1}}^{scale}\sum_{m=n\text{+1}}^{scale}{\text{sim}}_{\cos}\left({\overset{-}{x}}_{n},{\overset{-}{x}}_{m}\right)}{\left(scale*(scale-1)\right)}$, where ${\text{sim}}_{\cos}$ is the cosine similarity between vectors ${\overset{-}{x}}_{n}$ and ${\overset{-}{x}}_{m}$, and scale accounts for the number of CCs considered for the calculation of the average STD value and is dependent on the chosen investigation scale. For the “global” investigation scale, we considered all the CCs extracted from a set of spatiotemporal connectomes. For the “functional system” scale, we considered all the CCs with at least 20% of brain regions labeled according to a certain functional system f. For the “nodal” scale, we considered all the CCs involving a specific brain region. Related Matlab and Python scripts are publicly available at https://github.com/jvohryzek/STconnectomics_dvlp (Vohryzek, 2019).

To assess the reliability of measurements (Xing & Zuo, 2018; Zuo, Biswal, & Poldrack, 2019; Zuo, Xu, & Milham, 2019), we recalculated the nodal SD and STD cortical map for four runs of the HCP dataset with 97 subjects (B. Chen et al., 2015; Zuo et al., 2014; Zuo & Xing, 2014). The results can be found in the Supporting Information.

## SUPPORTING INFORMATION

Supporting information for this article is available at https://doi.org/10.1162/netn_a_00111. Related Matlab and Python scripts are publicly available at https://github.com/jvohryzek/STconnectomics_dvlp (Vohryzek, 2019).

## AUTHOR CONTRIBUTIONS

Jakub Vohryzek: Conceptualization; Methodology; Visualization; Writing - Original Draft. Alessandra Griffa: Conceptualization; Supervision; Writing - Review & Editing. Emeline Mullier: Methodology; Visualization. Cecilia Friedrichs-Maeder: Formal analysis; Writing - Review & Editing. Corrado Sandini: Data curation; Project administration. Marie Schaer: Data curation; Project administration; Resources. Stephan Eliez: Funding acquisition; Supervision. Patric Hagmann: Conceptualization; Methodology; Supervision.

## FUNDING INFORMATION

Patric Hagmann, National Centre of Competence in Research (NCCR) of Swiss National Science Foundation, Award ID: 158776. Patric Hagmann, National Centre of Competence in Research (NCCR) of Swiss National Science Foundation, Award ID: 185897. Patric Hagmann, Swiss National Science Foundation, Award ID: 156874.

## TECHNICAL TERMS

Functional systems: Assembly of brain regions across the cortex that have similar functional specialization.
Structural connectivity: A set of physical/white matter connections among neural assemblies.
Spatiotemporal connectome: A temporal network composed of connections between neural elements that are simultaneously coactive at the same time or consecutive time and have a structural connection between them.
Connected components: Brain’s dynamic functional patterns across time and cortical space calculated from the spatiotemporal connectome.
Spatiotemporal diversity: A measure quantifying how similar the dynamic functional patterns are across time and cortical space.
System diversity: A measure quantifying the distribution of functional systems active in the dynamic functional patterns across time and cortical space.
