# Supplementary material for: Dynamic spatiotemporal patterns of brain connectivity reorganize across development
Source: Netw Neurosci. 2020 Feb 1;4(1):115–33. doi: 10.1162/netn_a_00111 (PMC7006876; doi:10.1162/netn_a_00111)
Supplement: Supplementary file 1 [file netn-04-115-s001.pdf]

Vohryzek, J., Griffa, A., Mullier, E., Friedrichs-Maeder, C., Sandini, C., Schaer, M., Eliez, S., & Hagmann, P. (2020). Supporting information for "Dynamic spatio-temporal patterns of brain connectivity reorganize across development." *Network Neuroscience*, *4*(1), 115-133. https://doi.org/10.1162/netn_a_00111

Supplementary Information for Dynamic spatio-temporal patterns of brain connectivity reorganize across development

Jakub Vohryzek[1], Alessandra Griffa[1,4], Emeline Mullier[1], Cecilia Friedrichs-Maeder[1,5], Corrado Sandini[3], Marie Schaer[3], Stephan Eliez[3], and Patric Hagmann[1,2]

[1] Department of Radiology, University Hospital Centre and University of Lausanne, Lausanne, Switzerland

Lausanne, Switzerland

[2] Signal Processing Lab 5 (LTS5), Ecole Polytechnique Fédérale de Lausanne

[3] Department of Psychiatry, University of Geneva School of Medicine, Geneva, Switzerland

[4] Dutch Connectome Lab, Department of Complex Trait Genetics, Centre for Neuroscience and Cognitive Research, Amsterdam Neuroscience, VU University, Amsterdam, the Netherlands

[5] Department of Neurology, Bern University Hospital, University of Bern, Switzerland

Subject Age and cohort information

Overall 122 scanning sessions were considered for this project from which further 21 scans were removed due to corrupted recordings or abnormal cognitive scores. Out of the 101 scans further 14 were removed due to motion related artefacts (more than 10 % of all the timepoints above FD > 0.4mm and DV > 25). For the analysis we considered 87 scans (69 subjects) ranging between the 6-33yo (median (inter-quartile range) = 16:2(9:0)) (Figure 1). With respect to the subjects, the analysis was carried out as follows: 1) the age group analysis (children: median (inter-quartile range) = 10.7(2.8), adults: median (inter-quartile range) = 24.3(6.7) was performed for 21 children and 23 adults. We chose only the first timepoint for the subjects that

Vohryzek, J., Griffa, A., Mullier, E., Friedrichs-Maeder, C., Sandini, C., Schaer, M., Eliez, S., & Hagmann, P. (2019). Supporting information for "Dynamic spatio-temporal patterns of brain connectivity reorganize across development." Network *Neuroscience*. Advance publication. https://doi.org/10.1162/netn_a_00111
had two datapoints in the group as not to be influenced by non-independent results, 2) the correlation analysis was performed for the 69 subjects (87 scans). To account for longitudinal datapoints we averaged the subjects with multiple datapoints. For the independent analysis on the longitudinal data please see below the section on Repeated Measure Correlation.

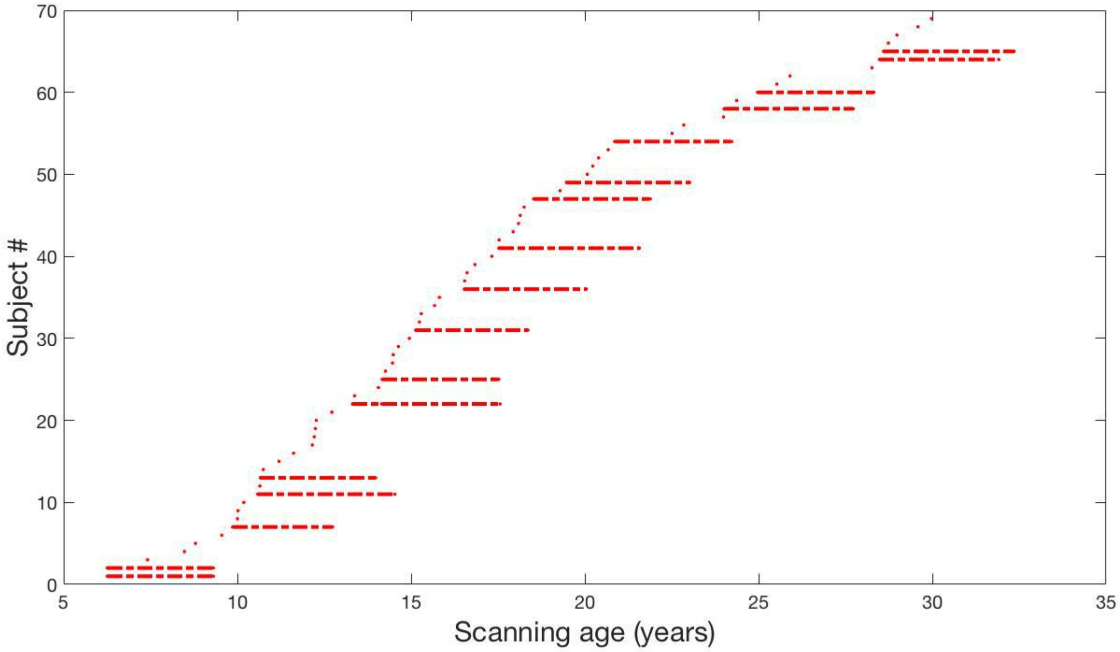

Figure 1: Subject Age: Age profile for all the subjects and their multiple longitudinal datapoints.

Information about the CCs

We calculated for every subject scan information about CCs yielded from the multilayer network. For the number of CCs each subject is part of, we found no correlation with age (r = -0.05, p-value =0.63). In terms of the spatial and temporal span of the CCs per subject we also found no significant differences with age. Spatial size (r = 0.16, p-value = 0.131) and Temporal size (r = 0.12, p-value = 0.27).

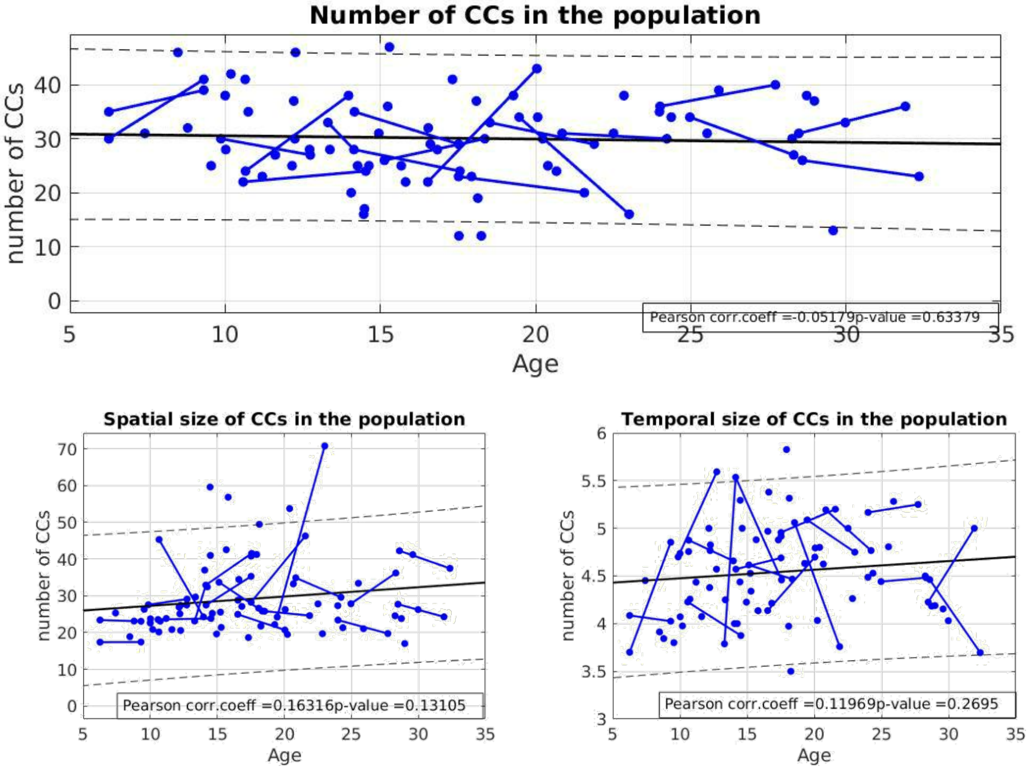

Figure 3: Spatial and Temporal profile of connected components for each subject with age. Spatial size (r = 0.16, p-value = 0.13) and Temporal size (r = 0.12, p-value = 0.27).

Construction of Spatio-temporal Connectome

Datasets

The spatio-temporal network was constructed according to (1). Briefly, as a functional dataset we used the individual subject BOLD timeseries for our 69 subjects (see above for details on subject age). The signal was pre-processed, z-scored and converted to binarised point-process at threshold of two standard deviation(1). As a structural dataset, we used the template structural connectivity matrix from the original DSI cohort of 68 subjects (1). We considered a robust structural connection if at least 50% of subjects possessed it. This approach and the

DSI dataset were chosen to minimise false negative and positive connections which are ultimately introduced through the white-matter tracts reconstruction (2).

Metadata

For the analysis on connection length, we used the original structural dataset of 69 subjects. We used the averaged connection length matrix across the subjects in order to label every edge in the multi- layer network with a length measure. To normalise for inter-subject head variability, we corrected the short and long edge-length threshold by a reference head size. To do so, we computed average InterCranial Volume (ICV) for a group of adult subjects (68 original subjects (1)) and used it to multiply thresholds for each subject as followed $\sqrt[3]{\frac{subject\ ICV}{adult\ group\ ICV}}$. Furthermore, for the inter-system edge analysis, we attributed to each cortical region a label with the associated functional system(3).

Motion Correction

In order to address motion-related confounds in the BOLD signal, we used quality measures, namely the Framewise Displacement (FD) and DV, and novel scrubbing approach in the network space. Firstly, we computed FD from six motion signals (three translations and three rotations). The rotational signal was converted from radians to millimetres (mm) as a displacement on a 50 mm radius sphere(4). On the other hand, Derivative of Variance (DV) was defined as the root mean square of the differentiated BOLD signal prior to pre-processing steps that might alter the signal such as nuisance parameters regression, linear detrending and bandpass filtering. We set the thresholds to FD=0.4mm and DV=25 and whenever corrupted timepoints exceeded 10% of the subject's recording timepoints for either FD or DV, the subject

was excluded. To be more rigorous, a novel scrubbing technique was performed in the graph space where we discarded all activations within the multilayer graph with spatial span of less than six regions and temporal span of less than two timepoints. Furthermore, we excluded all connected components implicated in corrupted timepoints and so eliminated any functional activations that might have arisen several points before and after the corrupted timepoints due to motion (Pearson's r = -0.239, p = 0.026)) Figure 2.

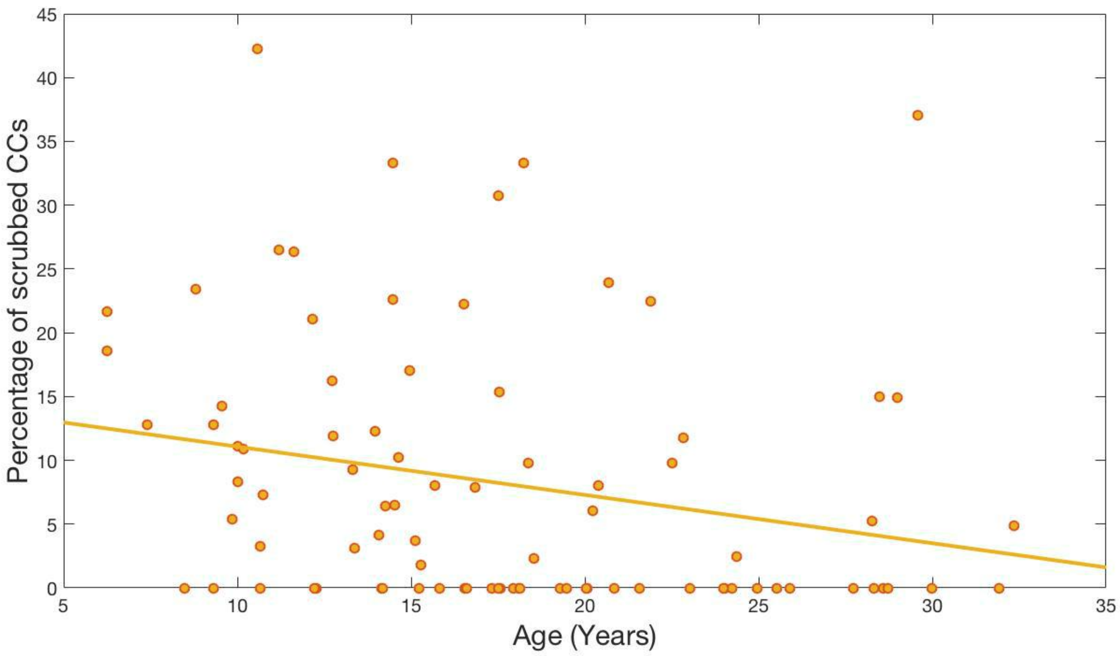

Figure 4: CC scrubbing: Percent of scrubbed CCs for every subject's scanning session (Pearson's r = -0.239, p-value = 0.026).

Repeated Measure Correlation

In addition to our main analysis we wanted to quantify intra-subject variability differences with age. To do so, we performed a repeated measure correlation (rmcorr). This method has been introduced to measure the intra-subject linear strength by removing the inter-subject variability and fitting the best parallel linear line to the individual subjects for varying

intercepts (5). For a subgroup of 17 subjects with repeated data acquisition with age. We found no significant results for the probability of short and long fibre usage with age (rmcorr = 0.173, p-value = 0.48 and rmcorr = 0.143, p-value = 0.55) and significant increase for the intra-subject variability for the proportion of inter-system edges with age (rmcorr = 0.569, p-value = 0.01).

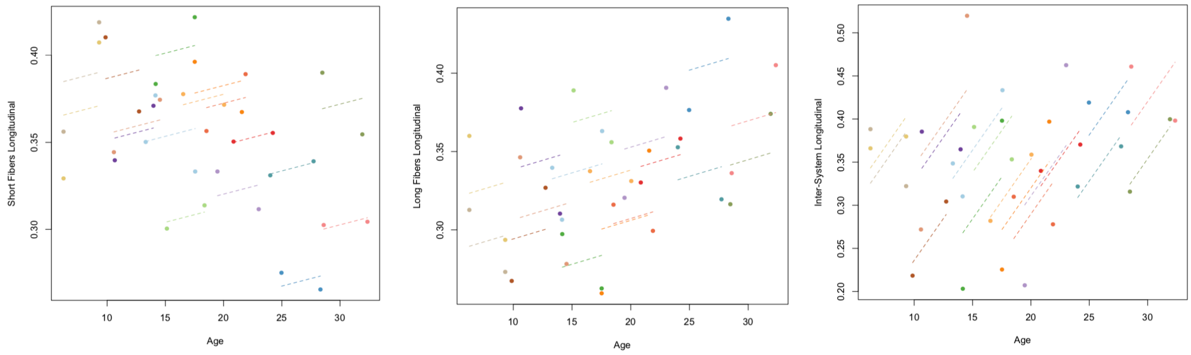

Figure 5: Repeated Measure Correlation, Intra-subject correlation of long and short edges was not significant (rmcorr = 0.173, p-value = 0.48 and rmcorr = 0.143, p-value = 0.55) (Left and Middle). Intra-subject correlation was significant for the inter-system edges with age (rmcorr = 0.569, p-value = 0.01) (Right).

Test-Retest Analysis

In order to address the reliability of the measures across different scanning sessions, we performed the same analysis on HCP dataset of 97 subjects. The resting-state fMRI dataset of each HCP subject consists of four runs of approximately 15 minutes each, with two sessions of two runs. Each session included oblique axial acquisitions alternated between phase encoding in right-to-left (RL) direction for run1 and phase encoding in left-to-right (LR) direction for run2. For every run, we calculated the nodal map of SD and STD from all the CCs of the 97 subjects. We compared all the sessions between each other using Pearson's correlation. The values can be found in Table 1. The correlation values between all pairs of

session were relatively high (minimum Pearson's correlation value = 0.9339), indicating a high reproducibility of SD and STD measures across different fMRI acquisitions, over the same set of subjects. To further verify the reproducibility of both measures, we plotted the SD and STD values on a standard cortical surface for all the four runs, as demonstrated in Figure 6 and 7. The cortical patterns obtained from different fMRI session were visually very similar.

	**SD**	S1(RL)	S1(LR)	S2(RL)	S2(LR)	**STD**	S1(RL)	S1(LR)	S2(RL)	S2(LR)
S1(RL)		1.0000	0.9339	0.9480	0.9318		1.0000	0.9774	0.9772	0.9756
S1(LR)		0.9339	1.0000	0.9313	0.9430		0.9774	1.0000	0.9755	0.9784
S2(RL)		0.9480	0.9313	1.0000	0.9295		0.9772	0.9755	1.0000	0.9775
S2(LR)		0.9318	0.9439	0.9295	1.0000		0.9756	0.9784	0.9775	1.0000

Table 1: Correlation between different scanning runs. The table shows Pearson's correlation between all the pairwise values for nodal System Diversity (SD) and Spatio-Temporal Diversity (STD) of the 97 HCP subjects. S1 and S2 represents the scanning sessions with RL being right-to-left and LR left-to-right phase encoding.

Vohryzek, J., Griffa, A., Mullier, E., Friedrichs-Maeder, C., Sandini, C., Schaer, M., Eliez, S., & Hagmann, P. (2019). Supporting information for "Dynamic spatio-temporal patterns of brain connectivity reorganize across development." Network *Neuroscience*. Advance publication. https://doi.org/10.1162/netn_a_00111

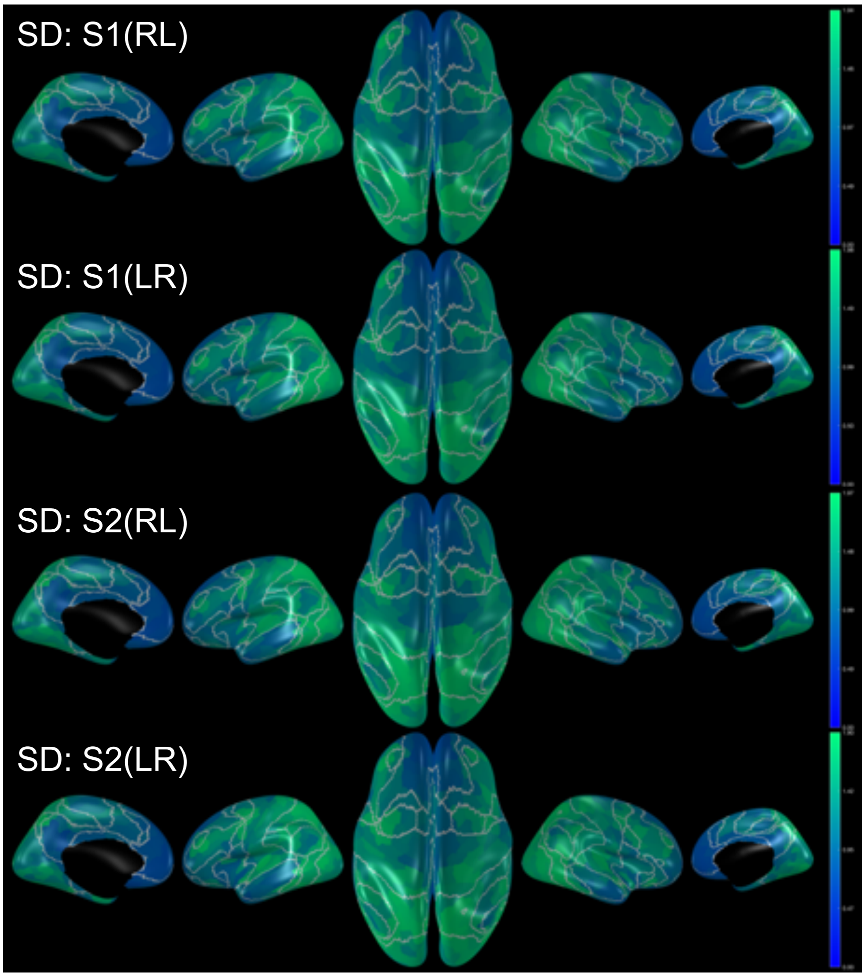

Figure 6: Brain cortical maps of System Diversity for four HCP runs. Correlation between different scanning runs. S1 and S2 represents the scanning sessions with RL being right-to-left and LR left-to-right phase encoding.

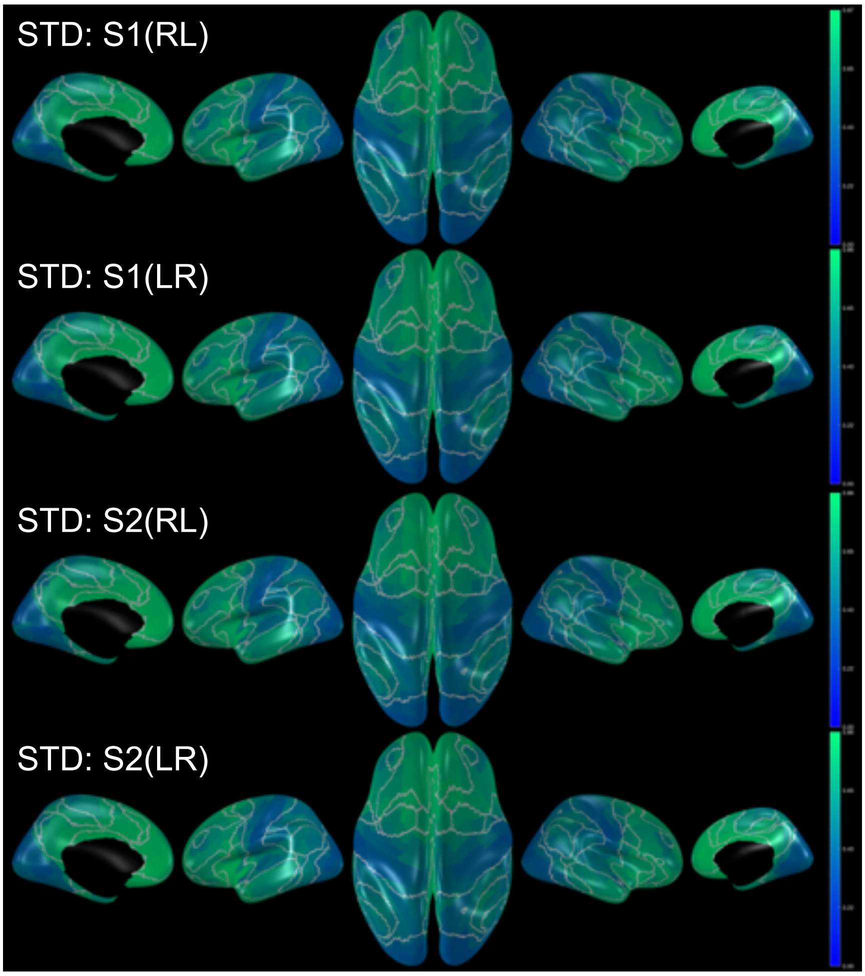

Figure 7: Brain cortical maps of Spatio-Temporal Diversity for four HCP runs. Correlation between different scanning runs. S1 and S2 represents the scanning sessions with RL being right-to-left and LR left-to-right phase encoding.

References

1. Griffa A, et al. (2017) Transient Networks of Spatio-temporal Connectivity Map Communication Pathways in Brain Functional Systems. *Neuroimage* (April):1–13.

2. de Reus MA, van den Heuvel MP (2013) Estimating false positives and negatives in brain networks. *Neuroimage* 70:402–409.

3. Yeo BTT, et al. (2011) The organization of the human cerebral cortex estimated by intrinsic functional connectivity. *J Neurophysiol* 106(3):1125–65.

4. Power JD, Barnes KA, Snyder AZ, Schlaggar BL, Petersen SE (2012) Spurious but

systematic correlations in functional connectivity MRI networks arise from subject motion. *Neuroimage* 59(3):2142–2154.

5. Bakdash JZ, Marusich LR (2017) Repeated measures correlation. *Front Psychol* 8(MAR):1–13.
